# Vaccine-Mediated Immune Responses to Experimental Pulmonary *Cryptococcus gattii* Infection in Mice

**DOI:** 10.1371/journal.pone.0104316

**Published:** 2014-08-13

**Authors:** Ashok K. Chaturvedi, Rumanasma S. Hameed, Karen L. Wozniak, Camaron R. Hole, Chrissy M. Leopold Wager, Susan T. Weintraub, Jose L. Lopez-Ribot, Floyd L. Wormley

**Affiliations:** 1 Department of Biology, The University of Texas at San Antonio, San Antonio, Texas, United States of America; 2 The South Texas Center for Emerging Infectious Diseases, The University of Texas at San Antonio, San Antonio, Texas, United States of America; 3 Department of Biochemistry, The University of Texas Health Science Center at San Antonio, San Antonio, Texas, United States of America; Geisel School of Medicine at Dartmouth, United States of America

## Abstract

*Cryptococcus gattii* is a fungal pathogen that can cause life-threatening respiratory and disseminated infections in immune-competent and immune-suppressed individuals. Currently, there are no standardized vaccines against cryptococcosis in humans, underlying an urgent need for effective therapies and/or vaccines. In this study, we evaluated the efficacy of intranasal immunization with *C. gattii* cell wall associated (CW) and/or cytoplasmic (CP) protein preparations to induce protection against experimental pulmonary *C. gattii* infection in mice. BALB/c mice immunized with *C. gattii* CW and/or CP protein preparations exhibited a significant reduction in pulmonary fungal burden and prolonged survival following pulmonary challenge with *C. gattii*. Protection was associated with significantly increased pro-inflammatory and Th1-type cytokine recall responses, *in vitro* and increased *C. gattii*-specific antibody production in immunized mice challenged with *C. gattii*. A number of immunodominant proteins were identified following immunoblot analysis of *C. gattii* CW and CP protein preparations using sera from immunized mice. Immunization with a combined CW and CP protein preparation resulted in an early increase in pulmonary T cell infiltrates following challenge with *C. gattii.* Overall, our studies show that *C. gattii* CW and CP protein preparations contain antigens that may be included in a subunit vaccine to induce prolonged protection against pulmonary *C. gattii* infection.

## Introduction

*Cryptococcus neoformans and Cryptococcus gattii*, the predominant etiological agents of cryptococcosis, are encapsulated fungal pathogens that cause life-threatening infections of the central nervous system (CNS) [1]. Cryptococcal meningoencephalitis is the most common disseminated fungal infection in AIDS patients [2]. Global estimates suggest that nearly one million cases of cryptococcal meningitis occur each year, resulting in approximately 625,000 deaths [3]. *Cryptococcus gattii* is traditionally considered to predominantly cause life-threatening fungal meningitis and infections of the lung and skin in otherwise healthy individuals [4]. However, *C. gattii* is now known to cause a significant proportion of opportunistic cryptococcal infections in HIV-infected individuals in sub-Saharan Africa [5], [6]. The geographical distribution of *C. gattii* was originally believed to be highly prevalent only in tropical and subtropical climates such as Australia, New Zealand, and Southeast Asia [7]. Nevertheless, *C. gatti* infections began to be detected within animal and human populations on Vancouver Island, British Columbia, Canada (a temperate climate) and the Pacific Northwest of the United States [6], [8]–[11]. Cryptococcosis due to *C. gattii* has also occurred in the Southwest, Southeast, and Northeast regions of the US and in Mediterranean Europe [6], [12]–[14]. Thus, individuals predicted to be at an exceptionally high risk for developing cryptococcosis (i.e., patients with compromised immune systems and immune competent persons residing in areas observed to contain *C. gattii*) represent ideal candidates for vaccination as a prophylactic measure.

Most studies to determine the protective immune response against pulmonary cryptococcossis have been performed using *C. neoformans*. The results of clinical and experimental investigations suggest that cell-mediated immunity by Th1- type CD4^+^ T cells is the predominant host defense response against cryptococcosis [15]–[17]. However, recent studies in mice suggest that host responses against *C. gattii* differ from those induced against *C. neoformans* [18]–[20]. In particular, *C. gattii* may exert a more suppressive impact on inflammatory responses compared to *C. neoformans*, which may partially explain the disparate clinical presentation of cryptococcosis induced by the two species.

*C. gattii* is categorized into four genotypes: VGI, VGII, VGIII, and VGIV, based on multilocus sequence typing (MLST) [21]. The VGII genotype of *C. gattii* is further divided into three subtypes: VGIIa, VGIIb, and VGIIc. *C. gattii* infections in the Vancouver Island outbreak were almost exclusively due to *C. gattii* strain R265 which is a member of the more virulent VGIIa genotype [22]–[24]. To date, there are currently no licensed vaccines available to prevent cryptococcosis and no protective *C. gattii*-specific antigens have been identified. While studies have evaluated the efficacy of various antigens to mediate protection against challenge with *C. neoformans* [25]–[35], studies examining vaccine-mediated immunity against *C. gattii* are limited. Importantly, it is essential to not assume that antigens demonstrated to be protective against *C. neoformans* will, likewise, induce protective immunity against *C. gattii*. The experiments described herein determined the efficacy of immunization with *C. gattii* protein preparations to induce protective immune responses against a lethal challenge with *C. gattii.* We show that vaccination of mice with *C. gattii* cell wall and/or cytoplasmic proteins results in significantly prolonged survival against experimental pulmonary challenge with *C. gattii* strain R265. Also, vaccination with *C. gattii* protein preparations results in the induction of pro-inflammatory cytokine and chemokine and Th1-type cytokine recall responses in splenocytes from vaccinated mice upon *C. gattii* antigen stimulation *in vitro*. The protein antigens revealed herein represent attractive candidates for the development of prophylactic sub-unit vaccines for the treatment and/or prevention of cryptococcosis due to *C. gattii* and perhaps *C. neoformans.*

## Materials and Methods

### Ethics

This study was carried out in strict accordance with the recommendations in the Guide for the Care and Use of Laboratory Animals of the National Institutes of Health. Mice were housed at the University of Texas at San Antonio Small Animal Laboratory Vivarium. These animal experiments were approved by The University of Texas at San Antonio Institutional Animal Care and Use Committee (IACUC), approved protocol number IS00000007, and mice were handled according to IACUC guidelines. All efforts were made to minimize animal suffering.

### Murine Model

Female BALB/c (H-2^d^) mice, 4 to 6 weeks of age (National Cancer Institute/Charles River Laboratories), were used throughout these studies. Mice were housed at The University of Texas at San Antonio Small Animal Laboratory vivarium and handled according to guidelines approved by the Institutional Animal Care and Use Committee. The mice were fed ad libitum and were monitored by inspection twice daily.

### Strains and Media

*C. gattii* strain R265 (VGII molecular genotype) was recovered from 15% glycerol stocks stored at −80°C. The strain was maintained on yeast extract peptone dextrose (YPD) agar (1% yeast extract, 2% peptone, 2% dextrose, and 2% bacto agar). Yeast cells were grown in YPD broth (Becton Dickinson and Company, Sparks, MD) for about 16–18 hours at 30°C with constant shaking. Yeast cells were collected by centrifugation and washed with sterile phosphate buffered saline (PBS) for further protein extraction. Quantification of viable yeast was performed using trypan blue dye exclusion in a hemocytometer for pulmonary infection.

### Protein Extraction

*C. gattii* strain R265 yeast was incubated overnight in liquid YPD medium at 30°C. The yeast were collected by centrifugation, washed twice in sterile PBS and divided into two fractions, one for the extraction of cell wall associated (CW) proteins as previously described [15] and the other for the isolation of cytoplasmic (CP) proteins. Cell pellets intended for the isolation of CW proteins were suspended in ammonium carbonate buffer, pH 8.4, containing 1% (v/v) β-mercaptoethanol (ME) and protease inhibitor cocktail (Roche Diagnostics GmbH, Mannheim, Germany), incubated for 45 min at 37°C with gentle agitation. After treatment, the cells were collected by centrifugation and the supernatant fluid sterile-filtered through 0.45-µM filters (Nalgene Nunc International Corp., Rochester, NY). The remaining cell pellet was suspended in YeastBuster Protein Extraction Reagent containing a protease inhibitor cocktail and tris(hydroxypropy)phosphine (THP) (Nalgene, San Diego, CA) according to the manufacturer's instructions and incubated for 45 min at 30°C with gentle agitation for extraction of CP proteins. After treatment, the cells were collected by centrifugation and the supernatant fluid containing CP proteins was filter-sterilized using a 0.45-µM filter (Nalgene Nunc International Corp.). The supernatants were then individually desalted and concentrated by centrifugation through an Amicon Ultrafree-15 (Millipore Corporation, Billerica, MA) centrifugal filter device. Protein content was estimated using the RC DC Protein Assay Kit (Bio-Rad, Hercules, CA). Subsequently, the proteins were further concentrated and non-protein contaminants removed using the ReadyPrep 2-D Cleanup Kit (Bio-Rad) according to the manufacturer's instructions.

### Immunization and Challenge Model

Separate groups of BALB/c mice were either mock-immunized with 50 µl of sterile endotoxin-free PBS (HyClone Lab. Inc., Logan, UT) or immunized with 50 µg of CW protein, 50 µg of CP protein, or a combination of CW and CP proteins (25 µg of CW and 25 µg of CP) in 50 µl of sterile PBS. Endotoxin content of the protein preparations were determined to be minimal (less than 1 EU/50 µg protein). Mice were immunized via intranasal inhalation because this is the most likely route of introduction of *C. gattii* into humans [1]. Mice were immunized three times, with four week intervals between each immunization. Ten days following the final immunization, mice received 1×10^4^ *C. gattii* by nasal inhalation as previously described [36]. Briefly, BALB/c mice were anesthetized with 2% isoflurane using a rodent anesthesia device (Eagle Eye Anesthesia, Jacksonville, FL) and then given a yeast inoculum of 1×10^4^ CFU of *C. gattii* strain R265 in 50 µl of sterile endotoxin-free PBS. The mice were fed ad libitum and were monitored by inspection twice daily. Survival was monitored daily, and mice that appeared moribund or not maintaining normal habits (grooming) were sacrificed.

Alternatively mice were euthanized on days 7, 14 and 21 post-*C. gattii* challenge. Prior to sacrifice, serum was collected by heart puncture into serum separator tubes (BD Microtainer, Franklin Lakes, NJ) from mice of each group. Serum was allowed to stand for 5 minutes in the serum separator tubes and then centrifuged at 6000 rpm for 5 minutes. After centrifugation, serum supernatants were carefully removed, aliquoted, and stored at −80°C for further use. Lung and spleen tissues were excised using aseptic techniques. The right lobes of the lungs were used to isolate pulmonary leukocytes whereas the left lobes of the lungs were processed for cytokine analysis as described below.

### Pulmonary Leukocyte Isolation

Lung tissues were excised on days 7, 14, and 21 post-infection, and subjected to enzymatic digestion at 37°C for 30 minutes in 10 ml of digestion buffer (RPMI 1640 and 1 mg/ml of collagenase type IV [Sigma Chemical Co., St. Louis, MO.]) with intermittent (every 10 minutes) stomacher homogenizations (Seward Stomacher 80, UK). The digested tissues were successively filtered through nylon filters (70 and 40 µm) (Fisher Scientific, Durham, NC) and washed with sterile Hank's Balanced Salt Solution (HBSS; Life Technologies, Grand Island, NY). This step enriches for the leukocyte population. Erythrocytes were lysed by incubation in NH_4_Cl buffer [0.859% NH_4_Cl, 0.1% KHCO_3_, 0.0372% Na_2_EDTA (pH 7.4); Sigma] for 3 minutes on ice followed by the addition of a 10-fold excess of PBS. The leukocytes were obtained after centrifugation (800×*g*) for 5 minutes, washing twice with sterile PBS, and suspending in sterile PBS + 2% heat-inactivated fetal bovine serum (FACS buffer). The cell count was determined using trypan blue dye exclusion in a hemacytometer. Flow cytometric analysis was used to determine the percentage of each leukocyte population as well as the absolute number of total leukocytes within the lung cell suspension for standardization of hemacytometer counts.

### Antibodies

For flow cytometry experiments, cells were incubated with CD16/CD32 (Fc Block) (BD Pharmingen Corp., San Diego, CA). The following antibodies conjugated to phycoerythrin (PE), allophycocyanin (APC), Alexa 647, or PECy7 were added to identify the subsets of leukocytes: CD45, CD11b/CD11c, (eBiosciences), F4/80 (Invitrogen), 1A8, and a cocktail of CD3, CD4 and CD8, (BD Biosciences).

### Flow Cytometry

To estimate the total leukocyte population, standard methodology using direct immunofluorescence method was employed. Briefly, in 96-well U-bottom plates, 100 µl containing 1×10^6^ cells in PBS + 2% FBS (FACS buffer) were incubated with 50 µl of Fc Block (BD Biosciences) diluted in FACS buffer for 5 minutes to block non-specific binding of antibodies to cellular Fc receptors. An optimal concentration of fluorochrome-conjugated antibodies were added in various combinations (between 0.06–0.5 µg/1×10^6^ cells) to allow for dual or triple staining and incubated for 30 min at 4°C. The cells were then washed three times with FACS buffer and fixed in 200 µl of 2% formaldehyde (Polysciences, Inc., Warrington, PA). Control wells were used to set the parameters for spillover and compensation calculations. Samples were analyzed using BD FACS Array software on a BD FACS Array flow cytometer (BD Biosciences). The absolute number of the total leukocytes was determined by multiplying the total number of cells calculated using the hemocytometer by the percent of CD45^+^ cells from flow cytometer. The absolute number of each leukocyte subset was determined by multiplying the percent of each subset by the total number of CD45^+^ cells.

### Cytokine Analysis

Cytokine levels in lung tissues were analyzed using the Bio-Plex Protein Array System (Luminex-based technology) Bio-Rad Laboratories, Hercules, CA). Briefly, lung tissue was excised and homogenized in ice-cold sterile PBS (1 ml). An aliquot (50 µl) was taken to quantify the pulmonary fungal burden and an anti-protease buffer solution (1 ml) containing PBS, protease inhibitors (inhibiting cysteine, serine, and other metalloproteinases) and 0.05% Triton X-100 was added to the homogenate. Samples were then clarified by centrifugation (800×g) for 5 minutes. The samples were centrifuged to remove cellular debris, and the supernatants aliquoted and stored at −80°C for further use. CFUs were quantified after 48 hours following incubation on YPD plates at 30°C. Briefly, the homogenized samples were assayed for the presence of cytokines including IL-1α, IL-1β, IL-2, IL-3, IL-4, IL-5, IL-6, IL-9, IL-10, IL-12 (p40), IL-12 (p70), IL-13, IL-17A, granulocyte colony stimulating factor (G-CSF), granulocyte monocyte colony stimulating factor (GM-CSF), interferon-γ (IFN-γ), CXCL1/keratinocyte-derived chemokine (KC), CCL2/monocyte chemotactic protein-1 (MCP-1), CCL3/macrophage inflammatory protein-1α (MIP-1α), CCL4/MIP-1β, CCL5/regulated upon activation, normal T cell expressed and secreted (RANTES) and tumor necrosis factor-α (TNF-α).

### Cytokine recall assay

Splenocytes (1×10^6^/well in 100 µl) from BALB/c mice immunized for 100 days with a combination of cell wall and cytoplasmic (CW/CP) proteins and splenocytes from naïve mice were cultured in RPMI complete media (Invitrogen, Grand Island, NY) supplemented with 10% heat-inactivated fetal bovine serum, 2 mM l-glutamine, 100 U penicillin/ml, 100 µg of streptomycin/ml, and 50 mM 2-mercaptoethanol (complete medium) with CW protein and CP proteins (50 µg each), hen egg lysozyme (HEL; 1 µg/ml) or concanavalin-A (Con-A; 1 µg/ml) as negative and positive controls, respectively. Endotoxin content of the protein preparations were determined to be minimal (less than 1 EU/50 µg protein). The samples were added to a 96-well plate and incubated for 24 hr at 37°C in 5% CO_2_. The culture supernatants were collected after 24 hr and mixed with 1 µl of 100X protease inhibitor (Roche) and then frozen at −80°C until cytokine expression was assayed using the Bio-Plex Protein Array System (Luminex-based technology; Bio-Rad Laboratories, Hercules, CA).

### Immunoglobulin isotyping

Immunoglobin isotypes were determined in serum using a commercially available kit (Zymed Laboratories, San Francisco, CA) according to manufacturer's instructions. We identified total antibody concentrations, antibodies specific for CW proteins, and antibodies specific for CP proteins at days 7 and 14 post-infection. Briefly, the wells of a microtiter plate (Corning Incorporated, Corning, NY) were coated with capture antibody designed to bind IgG, IgA, or, IgM. To measure *C. gattii*-specific CW and CP antibodies, 5 µg/ml of either CW or CP proteins in PBS was used for coating. The plates were then incubated overnight at 4°C, washed twice with PBS containing 0.05% Tween 20 (PBST) and blocked with PBS containing 1% BSA for 1 hour at room temperature. The sera from each group of mock-immunized, CW protein immunized, CP protein immunized, and combined CW and CP protein immunized mice were diluted 1 100 in PBST with 1% BSA and added to the wells. Plates were incubated for 2 hr at room temperature. The plates were washed with PBST and incubated with rabbit antibodies specific for the different mouse immunoglobulin subclasses: IgG_1_, IgG_2a_, IgG_2b_, IgG_3_, IgA and IgM for 2 hours at room temperature. After incubation, wells were washed with PBST and incubated with goat anti-rabbit IgG diluted 1∶250 in PBST with 1% BSA for 1 hour. Plates were then washed and developed with *o*-phenylendiamine substrate. Color development was stopped by addition of 100 µl per well of 1 M H_2_SO_4_, and the plate was read at 405 nm using a BioTek Elx 808 absorbance microplate reader with Gen5 v1.04.5 software (BioTek Instruments, Winooski, VT).

### Two-Dimensional Gel Electrophoresis

Immobilized pH gradient (IPG) strips (ReadyStrip IPG 11 cm, pH 4–7; Bio-Rad) were rehydrated in 200 µl of rehydration/sample buffer (Bio-Rad) containing 200 µg of the *C. gattii* CW or CP protein preparation. Isoelectric focusing (IEF) was carried out using PROTEAN IEF (Bio-Rad) under the following conditions: Step 1, 250 V for 20 min.; Step 2, ramped to 8000 V over 2.5 h, and Step 3, 8000 for a total of 30,000 V/h. Strips were then placed into equilibration buffer (Bio-Rad; 6 M urea, 2% SDS, 375 mM Tris-HCl pH 8.8, 20% glycerol, 2% DTT) for 15 min. Disulfide groups were subsequently alkylated by 10-min treatment with equilibration buffer of the same composition but using 2.5% w/v iodacetamide instead of DTT. Equilibrated IPG strips were then drained and placed on the top of 12.5% SDS-PAGE Criterion Precast Gels (Bio-Rad) and fixed using hot ReadyPrep Overlay agarose (Bio-Rad). The separation of proteins in the second dimension was carried out for 55 min at 200 V in Tris/glycine/SDS (TGS) running buffer (Bio-Rad) using Criterion electrophoresis equipment (Bio-Rad). Proteins in the gels were stained using SYPRO Ruby (Molecular Probes, Inc.) or, alternatively, transferred to PVDF membranes for immunoblot analysis.

### Immunoblot Analysis

Resolved proteins were transferred to Hybond-P polyvinylidene difluoride (PVDF) membranes (GE Healthcare, Buckinghamshire, UK) using a Semi-Dry Electrophoretic Transfer Cell (Bio-Rad) according to the manufacturer's instructions. The membranes were subsequently blocked using 5% non-fat milk in 20 mM Tris containing 500 mM NaCl (Tris-buffered saline) and 1% Tween 20 (TBS-T) for 1 h at room temperature. The blocking solution was then discarded and the membranes incubated overnight at 4°C with a 1∶200 dilution of immune sera collected on day 14 post-infection from mice immunized with CW and CP protein preparations. The membranes were then washed six times in TBS-T and antibody binding detected by the addition of goat anti-mouse IgG HRP-conjugated antibody (Pierce Biotechnology Inc., Rockford, IL) diluted 1∶1000 in TBS-T containing 5% non-fat milk for 1 h at room temperature. After six washes in TBS-T, the membranes were briefly incubated with SuperSignal West Dura Extended Duration Substrate (Pierce Biotechnology Inc.) and protein spots detected using a ChemiDoc XRS Camera and Quantity One 1-D analysis software (Bio-Rad).

### Identification of Proteins by HPLC-ESI-MS/MS

Individual spots of interest were excised manually under UV light from the gel using a sterile scalpel following 2-DE and digested *in situ* with trypsin (modified; Promega, Madison, WI). The digests were analyzed by capillary HPLC-electrospray ionization tandem mass spectra (HPLC-ESI-MS/MS) using a Thermo Fisher LTQ linear ion trap mass spectrometer fitted with a New Objective PicoView 550 nanospray interface. On-line HPLC separation of the digests was accomplished with an Eksigent NanoLC micro HPLC, column, PicoFrit™ (New Objective; 75 µm i.d.) packed to 10 cm with C18 adsorbent (Vydac; 218MS 5 µm, 300 Å); mobile phase A, 0.5% acetic acid (HAc)/0.005% trifluoroacetic acid (TFA); mobile phase B, 90% acetonitrile/0.5% HAc/0.005% TFA; gradient 2 to 42% B in 30 min; flow rate, 0.4 µl/min. MS conditions were: ESI voltage, 2.9 kV; isolation window for MS/MS, 3; relative collision energy, 35%; scan strategy, survey scan followed by acquisition of data dependent collision-induced dissociation (CID) spectra of the seven most intense ions in the survey scan above a set threshold. The MS datasets were searched against the NCBInr database [NCBInr 20130102 (22,378,659 sequences; 7,688,401,091 residues)] by means of Mascot (version 2.4.1; Matrix Science, London, UK). Methionine oxidation and cysteine carbamidomethylation were considered as a variable modification for all searches. Scaffold (Proteome Software, Portland, OR) was used to conduct an X! Tandem subset search of the Mascot data was followed by cross-correlation of the results of both searches. The Scaffold confidence levels for acceptance of peptide assignments and protein identifications were 95% and 99%, respectively.

### Statistical analysis

One-way analysis of variance (ANOVA) with the Tukey's post-hoc test was used to compare cytokine results using GraphPad Prism version 5.00 for Windows (GraphPad Software, San Diego, CA). Survival data were analyzed using the log-rank test (GraphPad Software). Significant differences were defined as *P*<0.05.

## Results

### *C. gattii* cell wall and cytoplasmic protein preparations induce partial protection against experimental pulmonary cryptococcosis

**Figure 1:**
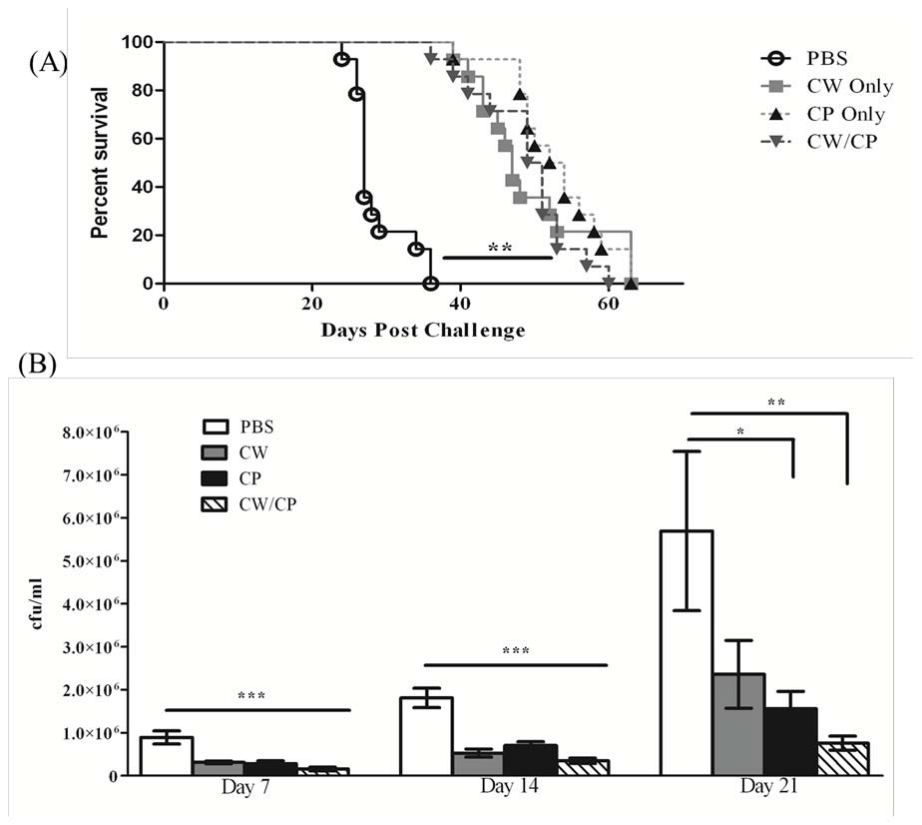
Protection afforded by immunization with *C. gattii* cell wall and cytoplasmic protein preparations. BALB/c mice were mock-immunized with endotoxin free PBS or immunized with total cell wall proteins (CW), cytoplasmic proteins (CP), or a combined CW and CP protein preparation (CW/CP) in 50 µl of sterile PBS by nasal inhalation. Mice were subsequently challenged with 10^4^ CFU of *C. gattii* strain R265 intranasally. Survival was monitored twice daily and mice that appeared moribund or not maintaining normal habits (grooming) were sacrificed (A). Survival data shown are cumulative of 2 experiments each using 8 and 6 mice per experimental group. Asterisk indicates significantly different (**P<0.001) survival rates for each immunized group compared to mock-immunized mice as assessed by the Log-rank (Mantel-Cox) test Alternatively, lungs were excised at days 7, 14, and 21 post-challenge and the pulmonary cryptococcal burden quantified (B). Pulmonary fungal burden data are cumulative of 3 experiments using 4 mice per time point. Results are expressed as mean CFU per milliliter ± SEM. Asterisks indicate where significant decreases (*P<0.05), (**P<0.001) and (***P<0.0001) were observed compared to mock-immunized mice.

BALB/c mice were immunized with *C. gattii* cell wall associated (CW) and/or cytoplasmic (CP) protein preparations or sterile endotoxin-free PBS (mock-immunized) as a control, as described in the Materials and Methods section. Ten days following the final immunization, mice were challenged with *C. gattii* strain R265 by nasal inhalation and survival (morbidity) monitored daily. Alternatively, mice were sacrificed on days 7, 14 and 21 post- *C. gattii* challenge to quantify pulmonary fungal burden. There was 100% mortality with a median survival time of 27 days in mock-immunized mice challenged with *C. gattii.* In contrast, mice immunized with CW proteins alone, CP proteins alone, or a combination of CW and CP proteins demonstrated significantly increased (*P*<0.05) median survival times of 47, 53, and 50 days, respectively, compared to mock-immunized mice (Figure 1A). Additionally, mice immunized with the individual CW or CP protein preparations alone or in combination showed a significant (*P*<0.05) reduction in pulmonary fungal burden compared to mock-immunized mice at days 7 and 14 post-challenge, while only mice immunized with CP or CW/CP proteins had significant reductions in fungal burden compared to mock-immunized mice at day 21 post-challenge (Figure 1B). The mice immunized with the combined CW and CP *C. gattii* protein preparation showed the highest reduction in pulmonary fungal burden compared to mock-immunized mice on each day observed. Brain fungal burden was also quantified on day 21 post-*C. gattii* challenge; however, no statistically significant differences in brain CFU between immunized compared to mock-immunized, mice were observed (data not shown).

### Splenocytes from immunized mice have increased cytokine recall responses to *C- gattii* proteins

We next evaluated cytokine recall responses in splenocytes from mice immunized with *C. gattii* CW and CP proteins. For this, mice were sacrificed ten days following the third immunization, and splenocytes were isolated from mock-immunized mice or mice immunized with both *C. gattii* CW and CP protein preparations according to the regimen provided in the Materials and Methods section. The splenocytes were then cultured in media alone, media containing *C. gattii* CW or CP protein preparations, or media containing either (HEL) or (Con A) as negative and positive controls, respectively, for 24 h and the supernatants collected for cytokine analysis. Significantly higher levels of IL-2, G-CSF, CXCL1 and IL-17A production were observed in splenocytes derived from immunized mice following CW stimulation (*P<0.05*) and significantly more IL-12p70, IL-1α, IL-1β, G-CSF, CCL2, CCL3, IL-6, CXCL1 and IL-17A production by splenocytes derived from immunized mice following CP stimulation compared to supernatants from splenocytes of mock-immunized mice (Table 1). A significant increase of Th2-type cytokines IL-4, IL-5 and IL-10 was also observed in culture supernatants of splenocytes isolated from immunized mice stimulated with CW proteins compared to splenocytes from mock-immunized mice (*P<0.05* in each instance). IL-10 production was significantly increased in culture supernatants of splenocytes from immunized mice stimulated with CP proteins alone compared to splenocytes from mock-immunized mice. Overall, the data shown in Table 1 indicate that immunization with *C. gattii* CW and CP proteins results in the induction of predominantly pro-inflammatory and Th1-type cytokine recall responses.

**Table 1:** Cytokine recall responses to *C. gattii* antigen^a^.

Cytokines	Con A Naive	Con A Immunized	HEL Naive	HEL Immunized	CW Naive	CW Immunized	CP Naive	CP Immunized
**Th1-type**								
IL-2	347.1±86.13	213.6±60.52	0.3760±0.15	0.3760±0.15	2.843±0.46	**93.27±6.11** *	2.372±0.68	57.72±23.75
IL-12p70	24.93±3.24	23.57±3.94	2.338±0.58	1.755±0.67	7.086±1.38	12.16±1.84	10.06±2.50	**22.00±3.70** *
IFN-γ	5.130±0.48	5.570±0.42	2.470±0.06	2.605±0.70	2.512±0.25	3.453±0.16	3.357±0.23	4.04±0.44
TNF-α	371.2±8.60	376.9±4.90	ND	ND	108.1±25.84	106.7±22.99	115.4±25.51	108.8±18.73
**Th2-type**								
IL-4	34.30±3.76	66.75±1.55	1.640±0.08	1.533±0.04	1.974±0.14	**10.78±0.33** *	6.018±2.08	5.731±1.54
IL-5	23.86±1.52	58.59±6.32	ND	ND	ND	**10.68±1.25** *	ND	1.458±0.33
IL-10	21.70±3.46	17.35±1.06	3.995±0.80	4.688±0.66	2.340±0.41	**13.23±0.76** *	2.200±0.47	**18.74±1.11** *
**Pro-inflammatory**								
IL-1α	6.028±0.98	3.368±0.30	0.4233±0.03	0.2750±0.17	1.788±0.17	3.195±0.33	4.223±0.52	**6.145±0.30** *
IL-1β	92.96±10.78	72.73±5.28	22.23±1.53	29.96±1.10	38.30±2.86	57.79±3.59	62.83±4.60	**87.11±4.28** *
G-CSF	5.405±0.96	3.353±0.84	ND	ND	17.55±2.13	**52.80±4.01** *	57.16±7.18	**122.0±10.16** *
GM-CSF	17.34±2.43	21.05±3.18	ND	ND	72.86±3.27	83.77±6.00	89.76±7.58	100.2±4.83
**Chemokines**								
CCL2/MCP-1	84.01±5.49	71.69±1.77	3.270±1.47	ND	6.652±1.30	15.53±1.92	13.11±1.90	**23.69±1.71** *
CCL3/MIP-1 α	294.0±51.51	154.9±22.69	16.55±2.75	22.21±2.47	64.80±4.54	118.4±8.61	160.2±12.94	**262.1±13.62** *
**Neutrophil associated**								
IL-6	40.22±6.36	24.24±3.04	1.393±0.21	2.308±0.08	1.403±0.40	5.895±0.34	2.792±0.34	**12.17±1.00** *
CXCL1/KC	9.708±1.08	9.598±1.95	4.403±0.62	2.713±0.73	50.53±3.83	**99.58±5.67** *	127.2±8.53	**210.2±10.98** *
IL-17A	41.79±7.44	84.42±10.70	0.4100±0.17	0.3550±0.20	0.798±0.21	**53.43±5.10** *	0.9683±0.11	**73.76±7.72** *

a Data shown are in pg/ml and are cumulative of three experiments using triplicate wells of each condition.

*Significance is *P<0.05* compared to mock immunized mice.

### Immunization with *C. gattii* proteins initiates early leukocyte recruitment

**Figure 2:**
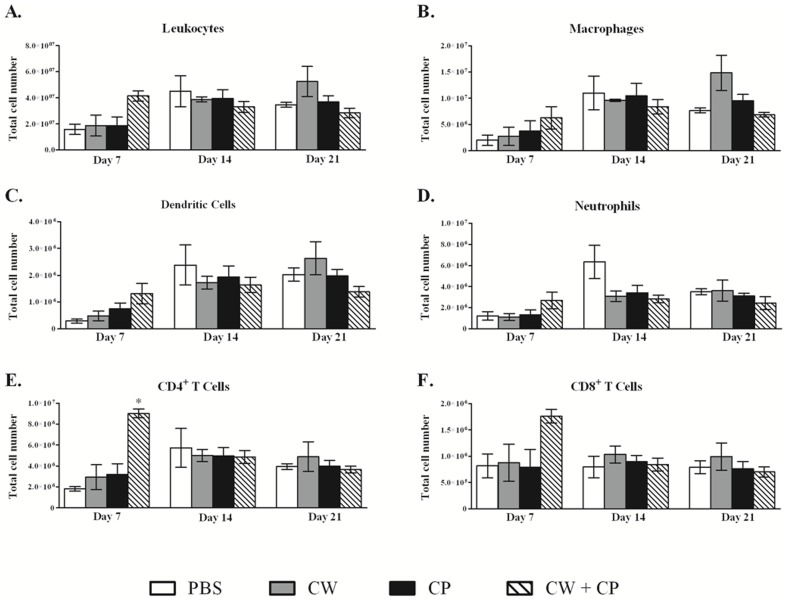
Pulmonary inflammatory leukocyte population at days 7, 14 and 21 post-infection. BALB/c mice were mock-immunized with endotoxin free PBS or immunized with total CW proteins, CP proteins, or a combined CW and CP protein preparation in 50 µl of sterile PBS by nasal inhalation. Mice were subsequently given an intranasal challenged with 10^4^ CFU of *C. gattii* strain R265. The lungs were excised at days 7, 14 and 21 post-inoculation and a single cell suspension generated using enzymatic digestion. Leukocytes were labeled with antibodies against CD45^+^ leukocytes (A), F4/80^+^ macrophages (B), CD11c^+^/CD11b^int^ DCs (C), 1A8^+^ neutrophils (D), CD3^+^/CD4^+^ T cells (E), and CD3^+^/CD8^+^ T cells. Data shown are mean ± SEM of absolute cell numbers from three separate experiments using 4 mice per group per experiment. Asterisks (*) indicate significant increases (*P*<0.05) in leukocyte populations compared to the mock-immunized mice with PBS (control). PBS immunized  =  clear bars, CW immunized  =  gray bars, CP immunized  =  black bars, CW/CP immunized  =  hashed bars.

Pulmonary leukocyte recruitment was then compared for mock-immunized mice and mice immunized with the various *C. gattii* protein preparations on days 7, 14, and 21 post-challenge. Recruitment of CD4^+^ T cells to the lungs of mice immunized with the CW and CP protein combination was significantly increased (*P<0.05*) at day 7 post-*C. gattii* inoculation compared to mock-immunized mice (Figure 2), but these differences were not observed at days 14 and 21 post-challenge. In addition, although not significant, the total number of CD8^+^ T cells was also increased in the combined CW and CP protein immunized group at day 7 post-challenge compared to mock-immunized mice (Figure 2). Interestingly, although each immunized group of mice survived significantly longer than mock-immunized mice (Figure 1A), no significantly increased trafficking of most leukocyte sub-populations into the lungs was observed compared to mock-immunized mice, particularly at the later time points post-challenge.

### *C. gattii* protein-specific antibodies from the serum of immunized mice

**Figure 3:**
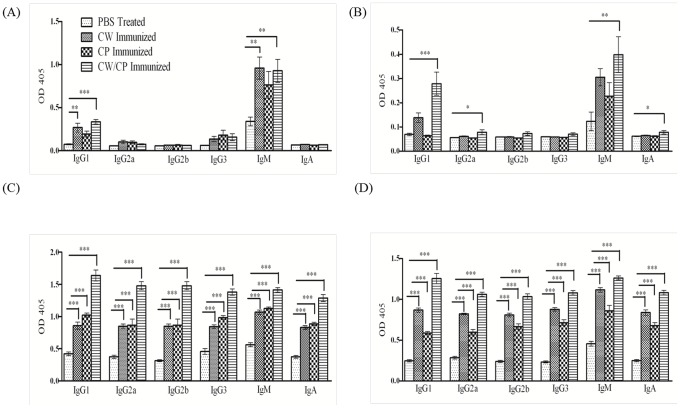
Evaluation of *C. gattii* specific antibody isotypes in mice during experimental pulmonary cryptococcosis. BALB/c mice were mock-immunized with endotoxin free PBS or immunized with total CW proteins, CP proteins, or a combined CW and CP protein preparation in 50 µl of sterile PBS by nasal inhalation. Mice were immunized three times, with four week intervals between each immunization. Ten days following the final immunization, mice received an intranasal inoculation of 10^4^ CFU with *C. gattii* strain R265. Serum was obtained from mock-immunized mice and mice immunized with CW or CP proteins or a combined CW and CP protein preparation on days 7 and 14 post-secondary challenge. The isotypes of *C. gattii* CW-specific antibodies on day 7 (A) and CP-specific antibodies on day 7 (B) and the isotypes of *C. gattii* CW-specific antibodies on day 14 (C) and CP-specific antibodies on day 14 (D) were determined. Asterisks indicate significant increase (*P<0.05), (**P<0.001) and (***P<0.0001) in antibody isotypes compared to the mice immunized with PBS (control). The results shown are cumulative of three experiments using 4 mice per experiment.

Serum obtained from mock-immunized mice or mice immunized with *C. gattii* protein preparations consisting of CW and/or CP proteins on days 7 and 14 following pulmonary challenge with *C. gattii* strain R265 were tested for the relative distribution of total immunoglobulin (Ig) isotypes: IgG_1_, IgG_2a_, IgG_2b_, IgG_3_, IgM, and IgA by ELISA. Also, the relative distribution of *C. gattii*-specific antibodies was determined using a *C. gattii* CW or CP protein preparation as the antigen for capture of *C. gattii*-specific serum antibodies. Results showed no significant differences in total Ig subclasses among any of the groups tested (data not shown). We observed a significant increase in the relative amounts of *C. gattii*-specific IgG_1_ *(P<0.001)* and IgM *(P<0.001)* antibodies on day 7 post-infection in mice immunized with the *C. gattii* CW protein preparation compared to mock-infected mice (Figure 3A). Similarly, significantly increased relative quantities of *C. gattii*-specific IgG_1_ *(P<0.001)* and IgM *(P<0.0001)* antibodies were observed on day 7 post-infection in mice immunized with the combined CW and CP protein preparation compared to mock-immunized mice (Figure 3A). A significant increase in *C. gattii*-specific IgG_1_ *(P<0.001),* IgG_2a_ *(P<0.01),* IgM *(P<0.001)* and IgA *(P<0.01)* Ig isotypes was observed in serum from mice immunized with the combined CW and CP protein preparation, compared to mock-immunized mice, when using *C. gattii* CP proteins for antibody capture (Figure 3B). However, on day 14 post-infection the relative levels of each *C. gattii*-specific Ig isotype tested in serum from all immunized groups were significantly higher *(p<0.001)* compared to the *C. gattii*-specific antibodies detected in mock-immunized mice (Figure 3C and D). Taken together, the results indicate that mice immunized with CW and/or CP proteins produce a significant increase in *C. gattii*-specific antibody recall responses following pulmonary *C. gattii* infection.

### Pulmonary cytokine expression during experimental cryptococcosis in protected mice

To evaluate local cytokine responses, lung homogenates were prepared from lungs excised from all experimental groups on days 7, 14, and 21 post-*C. gattii* challenge. Homogenates were evaluated for the presence of Th1-type (IL-2, IL-12p40, IL-12p70, IFN-γ), Th2-type (IL-4, IL-5 and IL-10) and pro-inflammatory (IL-1α, IL-1β, TNF-α, and IL-17A) cytokines, and chemokines (CXCL1, CCL4, CCL2 and CCL5). We observed significantly increased production of IL-4 and IL-12p40 in lung homogenates derived from mice immunized with CP proteins alone or the combined CW and CP protein preparation (*P<0.05* in each instance) on day 7 post-challenge compared to mock-immunized mice (Table 2). IL-4 levels in lung homogenates derived from CW protein immunized mice were also significantly increased at day 21 post-challenge compared to mock-immunized mice. Also, significantly more IL-17A, CCL5 and CXCL1 production was observed in lung homogenates derived from mice immunized with the combined CW and CP protein preparation (*P<0.05* in each instance) on day 7 post-challenge compared to mock-immunized mice. In contrast, we observed significantly less production of IL-12p40, IL-12p70, IL-1α, IL-1β, IL-17A, CXCL1, CCL2 and CCL5 in the lungs as the infection progressed (Table 2). The induction of CCL5, a chemokine involved in T cell infiltration, observed in the lungs of the combined CW and CP protein immunized group at day 7 post-*C. gattii* infection correlated with the increased CD4^+^ and CD8^+^ T cell lung infiltrates observed in these mice at the same time point (Figure 2). The overall decrease in the production of putatively protective cytokine and chemokine levels at days 14 and 21 post-challenge in the lungs of immunized mice and the absence of a prominent leukocyte response suggests that the early immune response to *C. gattii* infection ultimately was not sufficient to effectively resolve or contain the infection.

**Table 2:** Cytokine expression during cryptococcal pulmonary infection^a^.

Cytokines	PBS D7	CW D7	CP D7	CW/CP D7	PBS D14	CW D14	CP D14	CW/CP D14	PBS D21	CW D21	CP D21	CW/CP D21
**Th1-type**												
IL-2	23.54±2.63	15.96±1.39	15.25±1.64	33.46±4.25	17.70±2.99	19.15±5.79	18.87±5.42	15.23±4.18	31.07±8.25	27.79±8.28	23.15±3.97	18.65±3.08
IL-12p40	16.42±1.49	29.76±1.32	**36.19±2.66** *	**48.92±7.50** *	175.0±17.9	**75.82±5.09** *	**80.51±6.75** *	**73.24±8.85** *	145.1±18.8	**52.65±4.49** *	**55.69±4.37** *	**55.78±4.25** *
IL-12p70	39.68±5.15	**25.06±2.77** *	**23.88±3.85** *	38.81±2.23	60.31±2.43	59.10±8.15	57.16±4.06	42.06±3.83	59.75±7.31	54.78±6.34	42.83±3.17	34.24±2.46*
**Th1-type**												
IL-4	13.55±5.46	59.98±9.84	**83.85±22.11** *	**71.32±16.12** *	101.0±7.75	110.1±18.69	170.8±34.28	75.43±16.25	65.35±5.35	**98.25±8.07** *	81.98±7.41	65.22±9.51
IL-5	25.81±5.75	42.66±9.72	53.73±19.64	37.36±9.43	43.73±16.75	31.68±9.25	28.93±5.51	22.93±12.85	18.06±6.37	20.46±7.17	9.118±3.59	6.610±2.48
**Pro-inflammatory**												
IL-1α	39.74 ±3.18	30.19±1.71	34.34±5.23	41.52±4.35	128.5±11.7	**70.80±7.35** *	**74.71±7.02** *	**49.01±4.60** *	99.95±7.83	**70.34±5.36** *	80.76±3.90	57.23±5.32*
IL-1β	307.3±43.53	190.3±13.97	219.3±41.05	344.0±42.22	941.3±125.5	**455.2±83.43** *	464.0±**58.87** *	**444.5±117.1** *	702.0±76.09	**466.5±66.71** *	**395.2±38.8** *	**378.1±38.46** *
TNF- α	121.4±12.32	85.54±11.70	**71.74±12.37** *	108.4±8.33	122.0±5.82	160.3±19.79	159.5±13.54	164.2±45.98	151.6±20.42	153.1±12.12	110.7±8.31	101.3±14.69
IL-17A	7.282±0.88	9.448±1.14	15.78±3.81	**25.14±7.24** *	18.50±2.24	5.826±0.94	5.843±0.939	24.74±15.01	6.916±0.758	**4.256±0.70** *	4.790±0.409	4.907±0.647
**Chemokines**												
CCL3/MIP-1α	96.66±13.74	95.45±8.34	109.6±19.75	136.8±19.86	366.8±27.20	**192.4±22.50** *	**241.3±22.60** *	**199.7±28.88** *	234.2±15.64	190.3±21.15	**138.2±8.64** *	**123.1±9.897** *
CCL2/MCP-1	244.2±40.81	201.6±17.88	195.0±30.01	287.7±46.04	870.1±70.64	**401.2±44.94** *	**525.6±46.96** *	**303.1±41.22** *	367.1±55.87	517.5±88.32	316.2±31.96	257.5±55.34
CCL5/RANTES	252.5±22.87	354.3±29.68	287.4±34.22	**380.4±35.40** *	343.9±24.12	363.6±34.11	391.0±20.90	440.5±31.75	252.3±23.99	241.2±16.85	226.7±9.09	215.7±29.00
CXCL1/KC	189.1±25.97	192.2±17.24	251.8±49.42	**331.5±41.02** *	426.0±32.98	**283.4±28.34** *	330.0±36.23	**264.5±25.89** *	167.7±10.78	167.6±18.50	148.2±22.88	169.6±14.99

a Cytokine data are in pg/ml and cumulative of three separate experiments using 4 mice per group.

*significance is *P<0.05* compared to mock-immunized mice.

### Detection and identification of *C. gattii* immunodominant protein spots using immune sera from immunized mice

**Figure 4:**
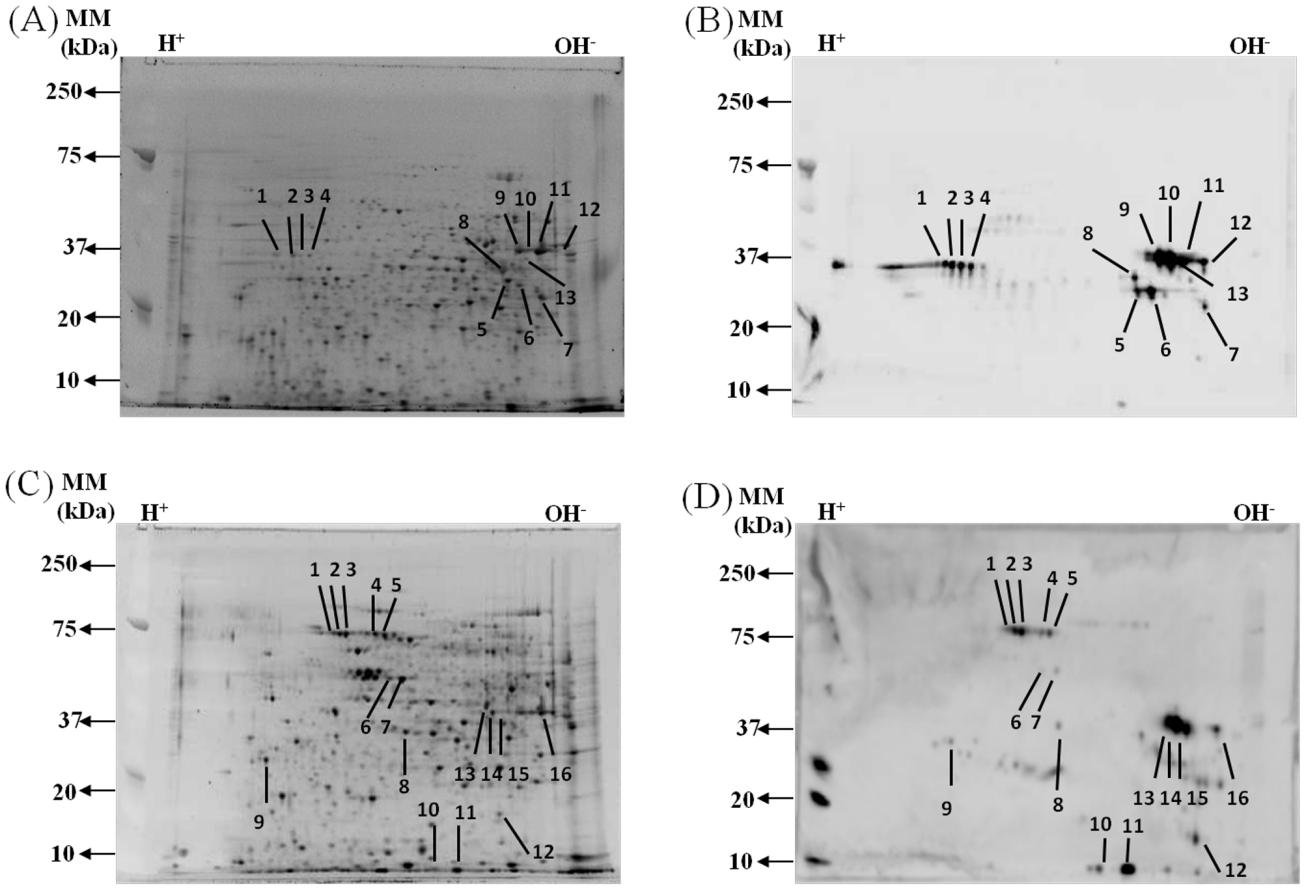
2-DE profile and immunoblot analysis of *C. gattii* cell wall and cytoplasmic proteins. Protein components were separated in the first dimension using a *pI* 4-7 IPG (11cm) strip and in the second dimension using a 12% polyacrylamide gel. Following 2-DE, the proteins were stained with SYPRO-Ruby Red. (A) CW protein profile; (C) CP protein profile. Proteins in separate gels were transferred to PVDF membranes and incubated with pooled serum taken on day 14 post-challenge of mice immunized using a combination of CW and CP *C. gattii* proteins: (B), CW immunoblot; (D), CP immunoblot. Numbered spots represent immunogenic proteins whose corresponding identities are given in Tables 3 and 4.

CW and CP protein preparations of *C. gattii* strain R265 were separated by 2-DE and analyzed for reactivity to serum by immunoblotting. After 2-DE, the gels were stained for total protein profile (Figure 4A and C) with SYPRO Ruby or alternatively transferred to PVDF membranes for immunoblot analysis using immune serum collected on day 14 post-challenge from mice immunized with a CW and CP protein combination. The immunoblot analysis was used as a way to identify potentially immunogenic cryptococcal proteins. Protein spot selection was determined following performing three biological replicates. CW protein immunoblot analysis detected a total of thirteen distinct protein spots (Figure 4B), whereas, CP protein immunoblot analysis detected a total of sixteen protein spots (Figure 4D). Each immunoreactive protein spot was subsequently excised from a parallel SYPRO Ruby-stained gel and the subsequent tryptic digest analyzed by HPLC-ESI-MS/MS. A summary of the identified immunoreactive proteins is provided in Tables 3 and 4.

**Table 3:** Immunodominant *C. gattii* cell wall proteins identified by HPLC-ESI-MS/MS after 2-DE.

SpotNo.	Protein Name^a^	MW (kDa)^b^	Accession Number^c^	Unique Peptides^d^	Sequence Cov. (%)
	Cell wall proteins				
1	Phosphopyruvate hydratase	48	XP_569379	6	19
2	Phosphopyruvate hydratase	48	XP_569379	8	26
3	Phosphopyruvate hydratase	48	XP_569379	11	31
4	Phosphopyruvate hydratase	48	XP_569379	10	29
5	Transaldolase	35	XP_003196531	12	32
5	Aldo-keto reductase	34	XP_003193262	5	11
6	Hypothetical protein CGB_B1480C	31	XP_003191973	3	7
7	Hypothetical protein CGB_C6700C	30	XP_003192954	3	26
7	Ribose-5-phosphate isomerase	31	XP_003196321	3	11
8	Transaldolase	35	XP_003196531	11	30
9	Hypothetical protein CGB_M1360W	34	XP_003197248	3	6
9	D-lactaldehyde dehydrogenase	38	XP_003194352	3	8
9	Zinc-binding dehydrogenase	38	XP_003197291	5	15
10	Hypothetical protein CGB_M1360W	34	XP_003197248	5	13
11	Mannitol-1-phosphate dehydrogenase	45	XP_003195433	5	12
12	Mannitol-1-phosphate dehydrogenase	45	XP_003195433	4	12
13	Aldehyde reductase	37	XP_003196615	3	9

a All proteins were identified with 100% probability in Scaffold.

b Molecular weight of NCBInr database entry.

c Accession number of NCBInr database entry.

d Peptides assigned with ≥95% confidence in Scaffold.

**Table 4:** Immunodominant *C. gattii* cytoplasmic proteins identified by HPLC-ESI-MS/MS after 2-DE.

Spot No.	Protein Name^a^	MW (kDa)^b^	Accession Number^c^	Unique Peptides^d^	Sequence Cov. (%)
	Cytoplasmic proteins				
1	Heat shock protein 70	69	XP_003192735	22	42
2	Heat shock protein 70	69	XP_003192735	25	43
3	Heat shock protein 70	69	XP_003192735	25	43
4	Heat shock protein 70	69	XP_003192735	11	23
5	Heat shock protein 70	69	XP_003192735	12	23
6	Phosphopyruvate hydratase	48	AFR93622	5	16
7	Phosphopyruvate hydratase	48	AFR97782	12	32
8	6-phosphogluconolactonase	40	XP_003194407	8	21
9	Mitochondrial precursor	32	XP_003194323	4	16
10	Hypothetical protein CGB_M1040C	15	XP_003197291	4	36
11	Hypothetical protein CGB_M1040C	15	XP_003197291	8	48
12	Translation initiation factor 5a	17	XP_003196060	2	19
13	Mannitol-1-phosphate dehydrogenase	45	XP_003195433	6	13
14	Mannitol-1-phosphate dehydrogenase	45	XP_003195433	4	11
15	Mannitol-1-phosphate dehydrogenase	45	XP_003195433	4	9
16	Oxidoreductase	33	XP_003193567	2	7

a All proteins were identified with 100% probability in Scaffold.

b Molecular weight of NCBInr database entry.

c Accession number of NCBInr database entry.

d Peptides assigned with ≥95% confidence in Scaffold.

## Discussion

*C. gattii* can cause disease ranging from mild to severe pneumonia to life-threatening fungal meningoencephalitis in otherwise healthy individuals [4]. However, *C. gattii* was shown to also cause a significant proportion of cryptococcal infections in HIV-positive persons in sub-Saharan Africa. Nonetheless, there is a paucity of published studies that evaluate vaccine-mediated immunity against pulmonary cryptococcosis caused by *C. gattii*. Consequently, the present study was undertaken to characterize vaccine-mediated immune responses against pulmonary *C. gattii* infection following intranasal immunization with *C. gattii* CW and/or CP protein preparations. We observed that mice immunized with *C. gattii* CW and/or CP protein preparations showed a significant reduction in pulmonary fungal burden during the earlier time points of the infection and significantly prolonged survival against challenge with *C. gattii* compared to mock-immunized mice. All mice eventually succumbed to *C. gattii* challenge most likely due to asphyxiation and not meningoencephalitis in keeping with clinical and experimental studies demonstrating that *C. gattii* infection typically does not cause fulminant meningoencephalitis upon pulmonary inoculation [8], [37]. While complete protection was not observed using our immunization protocol, these results are significant considering the morbidity and mortality associated with cryptococcosis due to *C. gattii* strain R265 that is observed both clinically and in experimental mouse models.

Most reported studies evaluating the role of antibody mediated immunity (AMI) during cryptococcosis have specifically targeted *C. neoformans.* Consequently, studies characterizing any role for AMI against *C. gattii* infections are lacking. We observed a significant increase in all Ig isotypes tested in serum of immunized, compared to mock-immunized, mice following pulmonary challenge with *C. gattii*. Previous investigations demonstrated that IgG isotypes IgG_1_, IgG_2a_ and IgG_2b_, but not IgG_3_, are protective against *C. neoformans* infection in mice [38]–[40]. Nonetheless, more definitive studies to evaluate the efficacy of passive transfer of serum from immunized mice or protection following challenge of immunized B cell knockout mice are needed to elucidate the efficacy of AMI against *C. gattii*.

Previous studies in our lab demonstrated that serum antibody generated in mice protected against pulmonary *C. neoformans* infection along with mass spectrometry analysis could be used to identify immunodominant cryptococcal proteins with the potential to induce protective anti-cryptococcal immune responses [15], [41]. Similarly, mass spectrometry analysis of the immunodominant proteins detected in our immunoblot studies revealed a number of proteins with undetermined function (i.e., hypothetical proteins) as well as proteins with known roles in stress response, signal transduction, carbohydrate metabolism, amino acid synthesis, and protein synthesis. Interestingly, some of the immunodominant proteins identified in our analysis of CW proteins (e.g., phosphopyruvate hydratase and mannitol-1-phosphate dehydrogenase) would be expected to be found in CP preparations. However, it is widely known that several cytosolic proteins are also associated with the cell walls of fungi [42]–[44].

The significant decrease in pulmonary fungal burden observed in mice immunized with CP proteins alone or in combination with CW proteins, but not mice immunized with CW alone, on day 21 post-challenge suggests that one or more proteins common to the CW and CP protein preparations, but more prevalent to the CP protein preparation, is responsible for the prolonged survival observed. Our mass spectrometry analysis identified phosphopyruvate hydratase and mannitol-1-phosphate dehydrogenase as immunodominant proteins that were present in both CW and CP protein preparations. Previous studies have shown that treatment of mice with recombinant enolase, also referred to as phosphopyruvate hydratase, conferred some protection against an experimental systemic challenge with *C. albicans* [45]. Also, phosphopyruvate hydratase was identified in previous immunoblot studies using serum from protectively immunized mice to identify immunodominant proteins of *C. neoformans* [41], [46]. These previous studies also identified heat shock protein (hsp) 70 in a *C. neoformans* CP protein preparation as immunogenic [41] which concurs with our findings herein. Hsp 70 is highly abundant and immunogenic *in vivo* during pulmonary cryptococcosis [47], [48], and heat shock proteins are highly abundant and immunogenic in other models of mycosis [49]–[53], as well. These findings support the inclusion of these proteins as components of a vaccine intended to induce protection against pulmonary cryptococcosis due to *C. gattii* and/or *C. neoformans.* Such a vaccine will be particularly important due to its broader clinical impact on the prevention of cryptococcosis in multiple patient populations and geographic settings.

While immunogenic cryptococcal antigens are often selected for analysis based on their serological activity [34], [46]–[48], proteins that are immunodominant for B cell epitopes may not necessarily be immunodominant for T cell epitopes. Previous studies have shown that vaccine-mediated immunity against pulmonary *C. neoformans* infection requires the induction of Th1-type CD4^+^ T cell mediated immune responses [reviewed in [54]]. Consequently, we elected to perform cytokine recall assays to determine cytokine responses, of immunized mice challenged with *C. gattii* antigens. Results of the cytokine recall assay suggested that immunization with either CW or CP protein preparations results in the induction of Th1-type cytokine (IL-2 and IL-12 p70), pro-inflammatory cytokine (IL-1α, IL-1β, IL-6, IL-17A and G-CSF) and chemokine (CCL2, CCL3 and CXCL1) production upon re-exposure to *C. gattii* proteins. Stimulation of splenic cells from immunized mice with CP proteins alone resulted in a greater induction of pro-inflammatory cytokines and chemokines while stimulation with CW alone resulted in an increase in non-protective Th2-type cytokine (IL-4, IL-5) production. These data suggest that immunization with the *C. gattii* CP protein preparation alone induces greater Th1-type and pro-inflammatory recall responses against *C. gattii* which may explain the lower fungal burden observed in mice immunized with CP proteins. However, analysis of cytokine profiles in the lungs of immunized, compared to mock-immunized mice demonstrated a gradual reduction in Th1-type cytokine (IL-2 and IL-12 p70), pro-inflammatory cytokine (IL-1α, IL-1β, IL-6, IL-17A and G-CSF) and chemokine (CCL2, CCL3 and CXCL1) production as the infection progressed. The lack of a sustained Th1-type and pro-inflammatory response observed *in vivo* is likely responsible for the lack of total protection observed in these studies considering that Th1-type cytokine responses are critical to the induction of protective immunity against *C. neoformans* [54]. This may also account for the lack of a cellular infiltration of leukocytes into the lungs to resolve infection. We observed a significant increase in the total number of CD4^+^ T cells as well as an increase (although not significant) in CD8^+^ T cells in the combined CW and CP protein immunized mice at day 7 post-challenge. However, this early increase in T cell infiltration in CW/CP-immunized mice was not sustained throughout infection. One hypothesis for the gradual reduction in the inflammatory response against *C. gattii* is that the yeast directly or indirectly suppresses host immune responses. Studies have shown that *C. neoformans*, a closely related species to *C. gattii*, produces components that down-modulate host immune responses including those of DCs and macrophages [reviewed in [18]–[20], [55]]. *C. gattii* has been shown to exert an even more suppressive impact on inflammatory responses than *C. neoformans* [18]–[20]. Nonetheless, the hypothesis that *C. gattii* suppresses host immunity does not fully explain why Th1-type and pro-inflammatory cytokine production in mock-immunized mice gradually increase until day 14 post-infection despite the mice having a significantly higher pulmonary fungal burden compared to immunized mice. More likely, Th1-type and pro-inflammatory cytokine responses in immunized mice are significantly lower compared to those observed in mock-immunized mice because the pulmonary fungal burden in the immunized mice is lower. Although significant reductions in pulmonary fungal burden and prolonged survival were observed in immunized mice, our results indicate that the amplitude and/or type (i.e., Th1-type) of recall immune response induced in immunized mice is insufficient to induce complete resolution of infection. Significantly better protection, compared to that observed herein, is likely to require the right combination of *C. gattii* antigens combined with an appropriate adjuvant to elicit complete protection against challenge. Subsequent studies to phenotype and mechanistically delineate vaccine-mediated immune responses against *C. gattii* infection can then be accomplished once more robust protection is generated.

In conclusion, we observed significantly prolonged survival against experimental pulmonary challenge with *C. gattii* strain R265 in mice vaccinated with *C. gattii* CW and/or CP protein preparations. Also, vaccination with *C. gattii* protein preparations results in the induction of pro-inflammatory cytokine and chemokine and Th1-type cytokine recall responses upon *C. gattii* antigen stimulation. However, the amnestic immune response induced by immunization with *C. gattii* CW and/or CP protein preparations alone was insufficient to induce complete protection against challenge. Nonetheless, the protein antigens identified in our study represent attractive candidates for the development of prophylactic sub-unit vaccines for the treatment and/or prevention of cryptococcosis due to *C. gattii* and perhaps *C. neoformans*.
